# Sarcomatous Tumour of the Shoulder-Centre in the Cortex of the Right Hemisphere

**Published:** 1895-06

**Authors:** J. E. Shaw, J. Paul Bush

**Affiliations:** Professor of Medicine in University College, Bristol; Physician to the Bristol Royal Infirmary; Surgeon to the Bristol Royal Infirmary


### SARCOMATOUS TUMOUR OF THE SHOULDER-CENTRE IN THE CORTEX OF THE RIGHT HEMISPHERE.

### OPERATION, IMPROVEMENT, DEATH.

BY

J. E. Shaw, M.B. Ed.,

*Professor of Medicine in University College, Bristol; Physician to the Bristol Royal Infirmary,*

AND

J. Paul Bush, M.R.C.S. Eng.,

*Surgeon to the Bristol Royal Infirmary.*

### OBSERVATIONS BY Dr. SHAW.

Although several cases of lesion of the cortical centre controlling the shoulder-joint have been diagnosed during life, and some few have been subjected to operation, it has seemed worth while to make the following case the subject of a brief record, inasmuch as it presented some rather unusual features—especially in relation to anæsthesia, amyotrophy, and the electrical reaction of the affected muscles. So far as the brain was concerned, the extent and position of the lesion were accurately determined at the times of the operation and of the *post-mortem* examination, thus increasing the value of the case from the physiological point of view.

E. C., æt. thirty-four, sailor, was admitted to the Bristol Royal Infirmary on January 25th, 1895, complaining of "fits." He has been a sailor twenty years; sixteen years ago he had yellow fever, but otherwise has had good health and no other illness except sore throats. He has had gonorrhœa and buboes, "which broke," but never constitutional syphilis. He has drunk heavily at times, but has touched no alcohol for five out of the last seven years. No history of injury to the head.

*History of Present Attack as given by himself.*—On December 9th last was on board his ship bound for Spain and he got up as usual in the morning at 8 a.m. He felt quite well, and had his breakfast; he then went to the "galley" with a book to read. He had just sat down when he felt a pain across the loins; this extended down the legs and up the body and all over him almost immediately. He says that his legs, arms, and neck became bent, and that he had cramps in them. Being unable to straighten them himself, his mates pulled them straight and kept them so. He did not lose consciousness, and he thinks the attack lasted twenty minutes. After the attack he felt stupid and light-headed. He did not pass urine or motions during the attack, after which he observed that his left arm and leg were weak. He was able still to walk a little and to move his arm; but when his attention was not attracted to the latter limb, it became bent up so that his hand touched the upper part of the chest. He afterwards became unable to walk, and felt very weak and ill. Upon December 15th he had another attack; this began with cramp in one leg (uncertain in which), and then almost suddenly extended throughout his body. There was the same sort of bending up of his body as in the first attack, and this one lasted three-quarters of an hour; he was fully conscious the whole time. On January 2nd, 1895, he had two more attacks; in these he had no cramps, but he felt giddy and said to a mate standing by that he was going to have another attack, and was thereupon carried to his cabin; consciousness was quite unaffected. He was sent to a hospital at Lisbon and stayed eighteen days, during which time he had four more attacks. After the one on January 2nd he noticed that he had lost all feeling in the left arm and leg. He could move his left arm about with his right hand, and was not able to perceive that it was being moved about. The sensibility gradually returned to some extent, but not completely. He has had pain in the head at times during the last three weeks; it starts in his forehead and then goes backwards. He has not had vomiting. He arrived at Southampton from Lisbon on the 23rd January, had no attacks during the voyage, but had one in the telegraph-office at Southampton the day before admission to the Infirmary.

*Present Condition.*—Patient is a well-nourished man, with his intelligence not obviously impaired. He complains of having had fits and of having partially lost power in the left side. There is a general loss of motor power in the left arm; but the shoulder-joint is more affected than the elbow, and this than the wrist and fingers. When seated in a chair he can move his fingers and grasp with them fairly; but he cannot raise his arm at all, and the pressure of a very few ounces is sufficient to prevent the limb from being abducted. When lying in bed he can raise his left leg from the bed and move his foot fairly well, but when standing it is found that his power of movement of the left hip-joint is very greatly impaired. The tongue when forcibly protruded deviates perhaps slightly towards the left; but no difference in movement, purposive or emotional, can be detected between the two sides of his face. The left knee-jerk is exaggerated, and ankle-clonus is easily obtainable; the left plantar reflex is absent. There is partial anæsthesia over the whole of the left side, face, arm, trunk, and leg, most marked about the shoulder, but nowhere is it complete. There is also partial analgesia over the same area. On percussion over the head, there is no tender spot to be discovered anywhere on the right side of the scalp. There are no signs of injury on the scalp, nor are there any scars or other signs about the body suggestive of syphilis. On examination of the fundus of each eye, in the left eye the papilla was seen to be slightly œdematous, but no positive neuritis; in the right eye there was distinct neuritis, with exudation and some small hemorrhages at the edge of the papilla. Vision $\frac{6}{6}$ in each eye. Upon examination with faradic current, it was found that there was a much diminished response in the muscles of the left side compared to those on the right; the left deltoid gave no reaction to the galvanic current. Patient does not vomit, and such headache as he has seems as much upon the left side of the head as the right. In the course of a few days it was found that patient's anæsthesia was very variable: at times it was almost absolute, even upon the left leg and side of the face, as well as in the left arm; at times also it was very slight, but was never quite absent. He was the subject also of paræsthesiæ, mainly restricted to the arm, but occasionally affecting the leg also: these consisted firstly of what he called a "hollow feeling" in the arm, which he had found to precede the fits (but which occurred frequently without being followed by a fit), and secondly subjective sensations of motion in the muscles of the leg and arm; it seemed to the patient that the limbs were moving from muscular spasm, when really the muscles were not contracting at all. At times also the posture-sense in the left arm was quite absent, so that he had to seek for his left arm under the bedclothes with his right hand. It was noticed also that motor paralysis varied somewhat in degree, and was found to be greater after an increase of the paræsthesia, although no spasm had occurred.

*February 1st.*—Patient vomited for the first and only time in his illness.

*February 3rd.*—Shortly before noon to-day patient had an attack of clonic spasm affecting the muscles moving the left shoulder-joint, and in a less degree those moving the left elbow-joint. The patient was fully conscious at the time, and called to those around him to observe what was happening: the arm was raised from under the bedclothes and was violently jerked about by alternate abduction and adduction of the shoulder, and some flexion and extension of the elbow; the wrist, fingers, and face were not affected, and apparently not the leg; the attack only lasted a few seconds.

The patient gradually became worse, both in impairment of mental power and increase of both motor and sensory paralysis. He suffered a good deal from bilateral headache; vomiting continued to be absent. The left side of the face became evidently paretic; the anæsthesia and analgesia became more marked and more persistent; power was almost entirely lost in the left hand; the right knee-jerk became exaggerated, and ankle-clonus could be obtained upon the right side; control over the bladder defective. The deficient response to faradic current was still more marked, but no abnormal sensitiveness to the galvanic current was developed. The pulse became slow, varying from 55 to 65. Upon admission it was observed that the muscles of the left shoulder (deltoid especially) and left upper arm were slightly atrophied; this wasting increased to a marked degree, the deltoid having nearly disappeared.

*February 22nd.*—The patient is so much worse in all respects, threatening to become comatose, that, without waiting longer for the appearance of further motor spasm, Mr. Bush was asked to operate upon him immediately.

### OBSERVATIONS BY MR. BUSH.

*February 22nd.*—The patient was placed under the influence of chloroform, and a large semi-circular incision was made on the right side of the head, beginning just above the level of the ear and passing upwards and backwards to within half-an-inch of the middle line above, and then forwards and downwards to a point one inch behind and one inch above the external angular process of the frontal bone, leaving the flap attached below. The flap so marked out was reflected downwards; a triangular area of bone was then removed by trephining the skull at three points and removing the intervening bridges of bone. The base of the triangle so formed was three inches long, parallel with and one inch distant from the interparietal suture ; the apex was over the centre of the fissure of Rolando, and its anterior and posterior edges were three inches in length. The dura mater was pulsating, and did not bulge unduly ; all hemorrhage having ceased, the skin-flap was replaced and held in position by four or five sutures ; the pulse-rate fell from seventy to thirty-eight per minute during the operation, which lasted over an hour.

*February 23rd.*—The patient passed a restless night, and there was some delirium; this morning he is quite rational, and answers questions with less effort than before operation. He has control over his bladder.

*February 24th.*—The patient says he is quite comfortable and in no pain ; the analgesia seems to be less marked ; there is no loss of the posture-sense of the left arm.

*February 26th.*—The paralysis of the left side of the face remains the same, the tongue still deviates to the left, anæsthesia and analgesia not so marked as they were ; there is ankle-clonus in the right leg, but the patellar reflexes are much less marked than before operation.

*February 27th.*—A quarter of a grain of morphia was injected subcutaneously, and ten minutes later the skin-flap made at the former operation was turned down. The dura mater was opened by a crucial incision, which exposed the upper part of the ascending frontal convolution and surrounding portions of cortex. The cortex was then stimulated with an interrupted electric current, which produced movements at one point only ; viz., immediately in front of and one inch below the upper end of the fissure of Rolando, clonic contractions of the left fore-arm resulting. The brain was incised at this spot and the subcortical tissue explored with a director, but nothing abnormal could be felt. Chloroform was then administered, and a $\frac{3}{4}$-inch trephine applied to the posterior superior angle of the triangle and a disc of bone taken out ; the opening thus made was connected with the original one by removing the intervening bridge of bone. The lower portion of the upper third of the ascending parietal convolution was thus exposed, without result ; but movements of the left arm were elicited by mechanical stimulation of this area. A free incision was then made into the subcortical tissue above and in front of the junction of the upper with the middle third of the fissure of Rolando, as in this position considerable bulging had taken place since the commencement of the operation, and a tumour-like mass was found, about an inch in diameter, which was of a peculiar bluish colour ; this was freely removed with the bowl of a tea-spoon, till the edge of what appeared to be healthy brain-tissue came into view ; the bleeding at this period of the operation was slight, two small vessels only requiring ligature on the surface of the brain ; the edges of the dura mater were brought together with fine catgut sutures, and the skin-flap carefully replaced, a small rubber drainage-tube being inserted between the dura mater and the scalp at the posterior edge of the wound; a stream of warm boracic lotion was kept almost continuously running over the exposed surface.

*Subsequent Report.*—The immediate result of the operative measures was distinctly beneficial. Some amount of voluntary motor power was regained in the muscles moving the left thigh; the anæsthesia was almost absent, and the intelligence was much improved. Within four days, however, nocturnal delirium set in, attended with a return of the various paralytic phenomena, the left side of the face becoming gradually more markedly paralysed than it had been at all before. The sensory paralyses, although they increased in degree, were observed to vary considerably from day to day.

OBSERVATIONS BY DR. SHAW.

During the last ten days of his life patient became gradually more stupid and apathetic, although he could recognise persons up to two days before his death, on March 13th. The left hemiplegia became complete, as also did the correlated anæsthesia and analgesia, even the left cornea becoming anæsthetic. The optic neuritis and retinal hemorrhages became much more marked. No further convulsion occurred, nor vomiting. A slight response to the Faradic current was obtainable in the paralysed muscles of forearm and lower leg not many hours before death.

*Autopsy.*—March 14th. No lesion of any special importance was found in the body, which had become rapidly emaciated before death. The brain having been removed, was set aside to harden in spirit. The incisions made at the time of the operation showed almost no evidence of reparative processes having taken place during the three weeks patient had lived. After a month's hardening, the brain was very carefully examined by Dr. Edgeworth and myself. A considerable mass of tumour was found: this new growth occupied the posterior end of the superior frontal convolution causing some bulging into the great longitudinal fissure; it had extended backward and downward into the ascending frontal convolution opposite the superior frontal sulcus and upper portion of the middle frontal convolution. We positively determined the fact that it was sharply limited posteriorly by the fissure of Rolando and did not invade the parietal convolution; we also carefully established the fact that the gyrus fornicatus and internal capsule on the right side were free from any lesion which might have caused the anæsthesia. The bulk of the whole original tumour would have been fully that of a Tangerine orange. It extended into the white substance below the cortex for quite half an inch. Microscopical examination of the tumour showed it to be a round-celled sarcoma with very numerous hemorrhages. The spinal cord was not examined.

*Remarks.*—With the exception of vomiting, the patient had the classical symptoms of cerebral tumour, but the confused history he gave of the "fits" he had had previous to admission did not do more than suffice to show that they were probably "Jacksonian," in type and depended almost certainly upon a lesion in the cortical or subcortical region of the right hemisphere. The great degree of paralysis of the left shoulder, and to a less extent of the left hip-joint, made it practically certain that the lesion corresponded in level to the second fourth (counting from above) of the fissure of Rolando, in front as well as probably behind that sulcus. The one convulsive attack which the patient had while under our observation proved that the shoulder centre was the site of the effective cortical lesion. It was probably the case, judging from the large size of the tumour, that it had originated in the superior frontal convolution, causing no symptoms; and when, by extension downwards and backwards, it reached the ascending frontal convolution, recognisable consequences were developed. The most curious part of the case, however, was undoubtedly the great degree of anæsthesia and analgesia which was present. The relation of the so-called excito-motor area to sensation seems as far from being settled as ever. Flechsig maintained[1] that the motor zone of the brain is the sensory centre of the posterior spinal columns; but many lesions of this zone have been met with, anæsthesia being

[1] *Neurol. Centralbl.*, vol. ix., 1890, p. 417.

absent. Curiously, for some unknown reason, this anæsthesia, when it is developed, generally is so after operative treatment has been carried out (*e.g.* Bremer and Carson's case[1]). These last-mentioned authorities consider that "so far as the middle third of the Rolandic region is concerned, the anterior portion is exclusively motor, the posterior chiefly sensory, in function." This view, however, our case does not corroborate, as the posterior portion of the Rolandic area was not involved. Everett Flood and Schäfer in their Report to the Scientific Grants Committee[2] give account of their experiments in removing portions of the Rolandic cortex in the Macacus monkey. The centres controlling the cheek-pouches of the animals were removed, which caused anæsthesia as well as motor paralysis of the pouch; it was also, however, observed that the anæsthesia was of complete hemiplegic distribution in some few of the cases, forming an analogy to our case, where at the time of the operation the shoulder-centre was as completely destroyed by the growth as if it had already been removed. The loss of muscular sense is recognised as a sensory symptom suggestive of cortical lesion, when not associated with tactile anæsthesia. An interesting example of this is recorded by Murray and Richardson[3]; here the deficiency of the posture-sense was much remedied by operation. On the other hand, Allen Starr and McCosh record a case[4] where temporary loss of posture-sense ensued immediately upon operation upon the parietal cortex.

In the absence of examination of the spinal cord in our case, it is not perhaps right to lay too much stress upon the muscular wasting and alteration of the electrical reactions. That these were due, however, to brain- and not cord-condition may be fairly inferred from the fact that they were most marked in the muscles supplied from the injured brain-centres. Many cases of cortical amyotrophy have been recorded: in most or all the arm has been paralysed either singly or in association with other parts. The diminished response both to the faradic

[1] *Am. J. M. Sc.*, vol. cix., 1895, p. 120.

[2] *Brit. M. J.*, 1894, vol. ii., p. 189. [3] *Lancet*, 1895, vol. i., p. 665.

[4] *Am. J. M. Sc.*, vol. cviii., 1894, p. 517.

and galvanic currents in our case would have been thought in past days to point necessarily to a "peripheral" lesion. We know, however, now that the old rules for diagnosing between a "central" and "peripheral" lesion by means of electrical tests are not universally applicable and true, and must not be too seriously regarded. Leszynsky[1] reviews the value of electricity in the diagnosis of peripheral lesions.

In conclusion, the case seems to show that the functions of the excito-motor area of the cerebral cortex are still imperfectly known.

[1] *Med. Rec.*, vol. xlvi., 1894, p. 199.

